# Supplementary material for: Place Cells in Head-Fixed Mice Navigating a Floating Real-World Environment
Source: Front Cell Neurosci. 2021 Feb 12;15:618658. doi: 10.3389/fncel.2021.618658 (PMC7906988; doi:10.3389/fncel.2021.618658)
Supplement: Supplementary file 1 [file Data_Sheet_1.pdf]

Supplementary Material

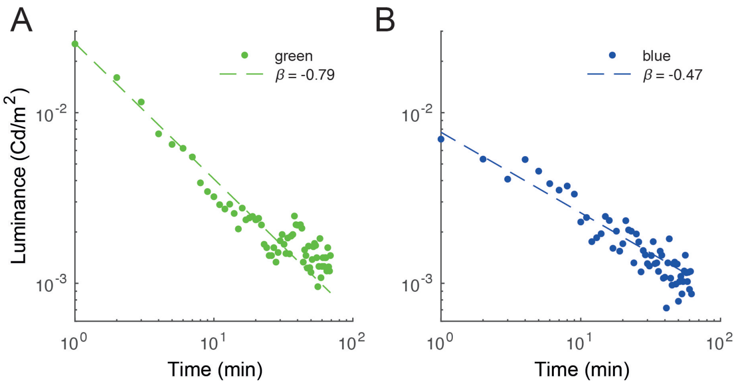

Figure S1. Luminance of phosphorescent tapes used as visual cues decays at a power law rate. Tapes were coloured (**A**) green (peak 520 nm) and (**B**) blue (peak 500 nm). Dashed lines denote curve fits of the form $y = ax^{\beta}$. The luminance over an imaging period of an hour is well within the sensitivity range for mice, within the scotopic (low-light) range (Umino et al., 2008).

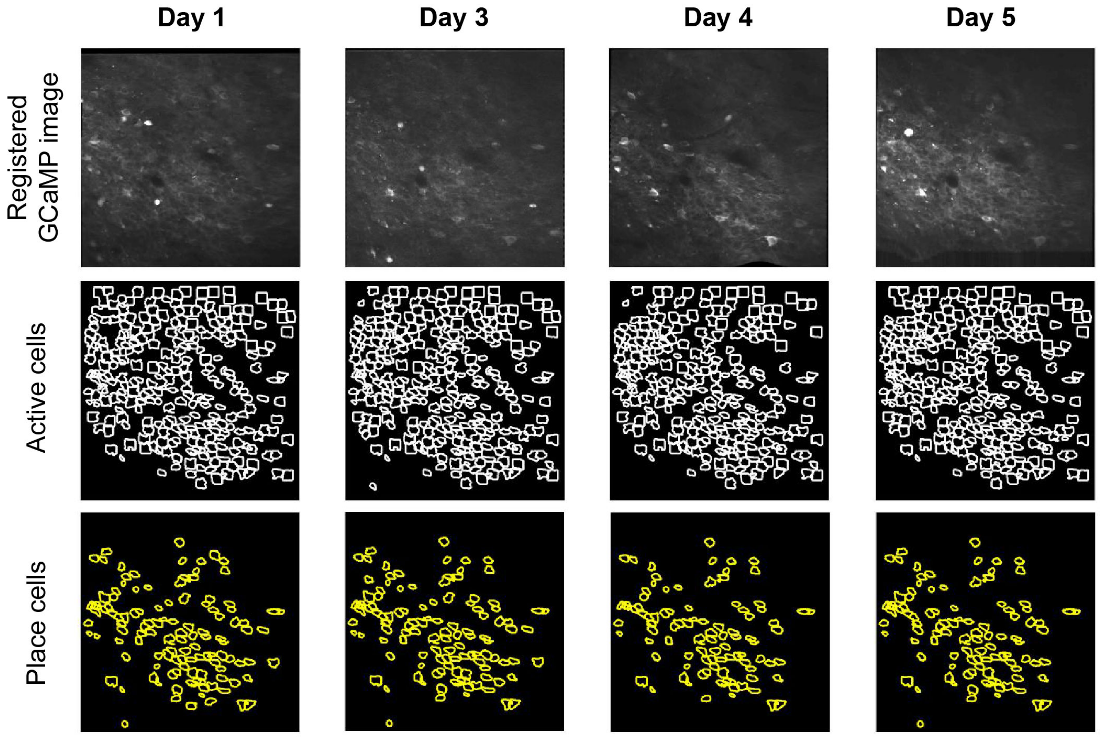

Figure S2. Image registration allows cells to be tracked across multiple imaging sessions. GCaMP images from different sessions were motion-corrected using the image from one session as template. The registered images (top row) were then temporally concatenated and the ROI segmentation algorithm was run on the concatenated video to produce a map of all the active cells. Active cells for each session (middle row) were identified based on the deconvolved neural activity for the session. The subset of active cells that were place-sensitive (bottom row) were then identified by place field analysis.

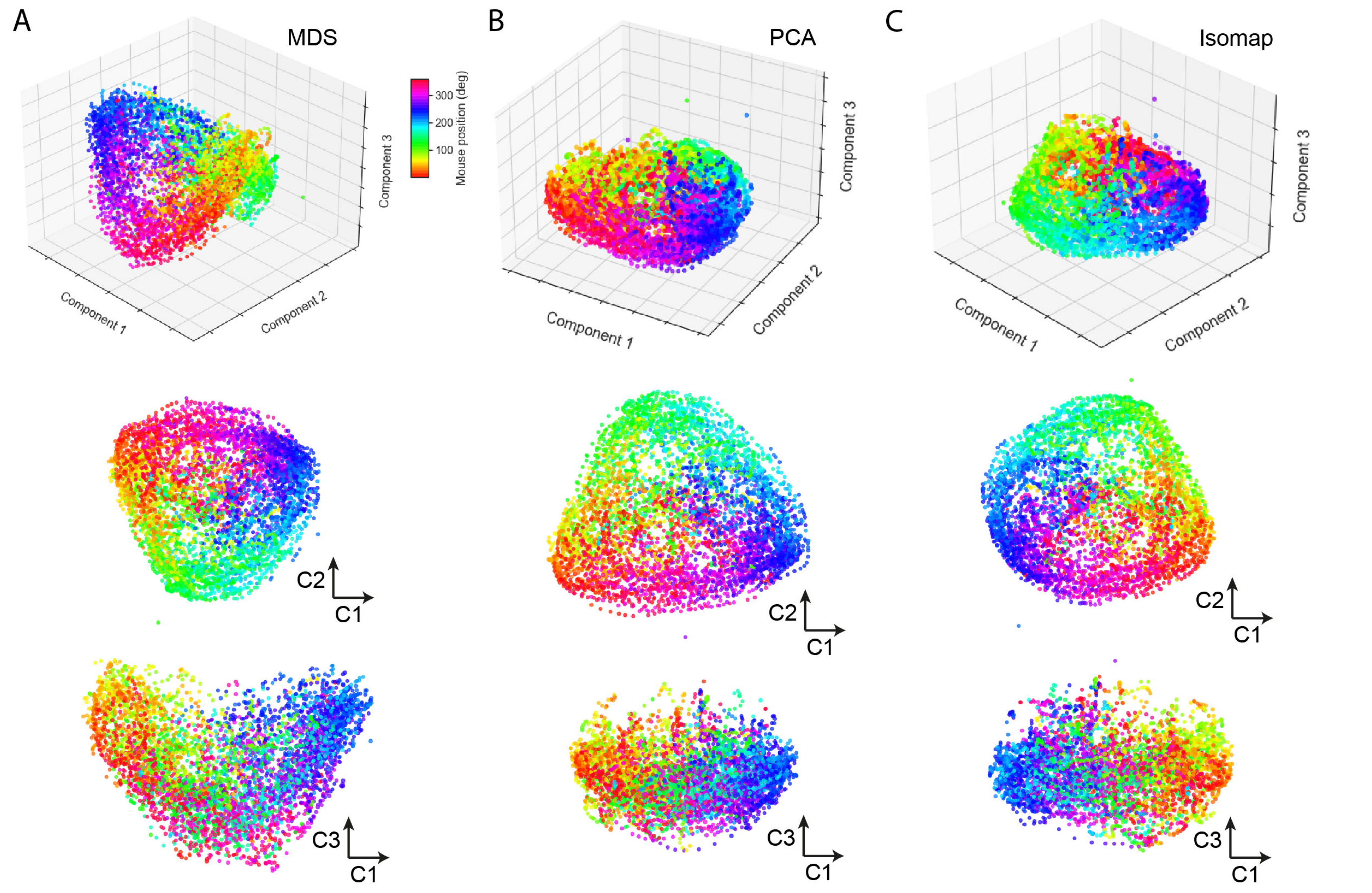

Figure S3. Neural manifolds extracted using (**A**) MDS, (**B**) PCA and (**C**) Isomap are very similar. Example shown here is the same circular track recording shown in the main manuscript. While MDS systematically captures at least as much of the variance as PCA (normally more) for the same number of dimensions, it also shows residual sructure related to mouse spatial position beyond the second dimension, which is not apparent in PCA or Isomap. Isomap provides a very similar depiction to MDS and PCA, although the "variance accounted for" quantification of manifold performance does not directly apply in this case. Our conclusion is that the precise technique used to visualise the manifold is not crucial.
